# Supplementary figures and images for: Immunomodulatory therapy with glatiramer acetate reduces endoplasmic reticulum stress and mitochondrial dysfunction in experimental autoimmune encephalomyelitis
Source: Sci Rep. 2023 Apr 6;13:5635. doi: 10.1038/s41598-023-29852-x (PMC10079956; doi:10.1038/s41598-023-29852-x)

**Full Length Western Blot Films**


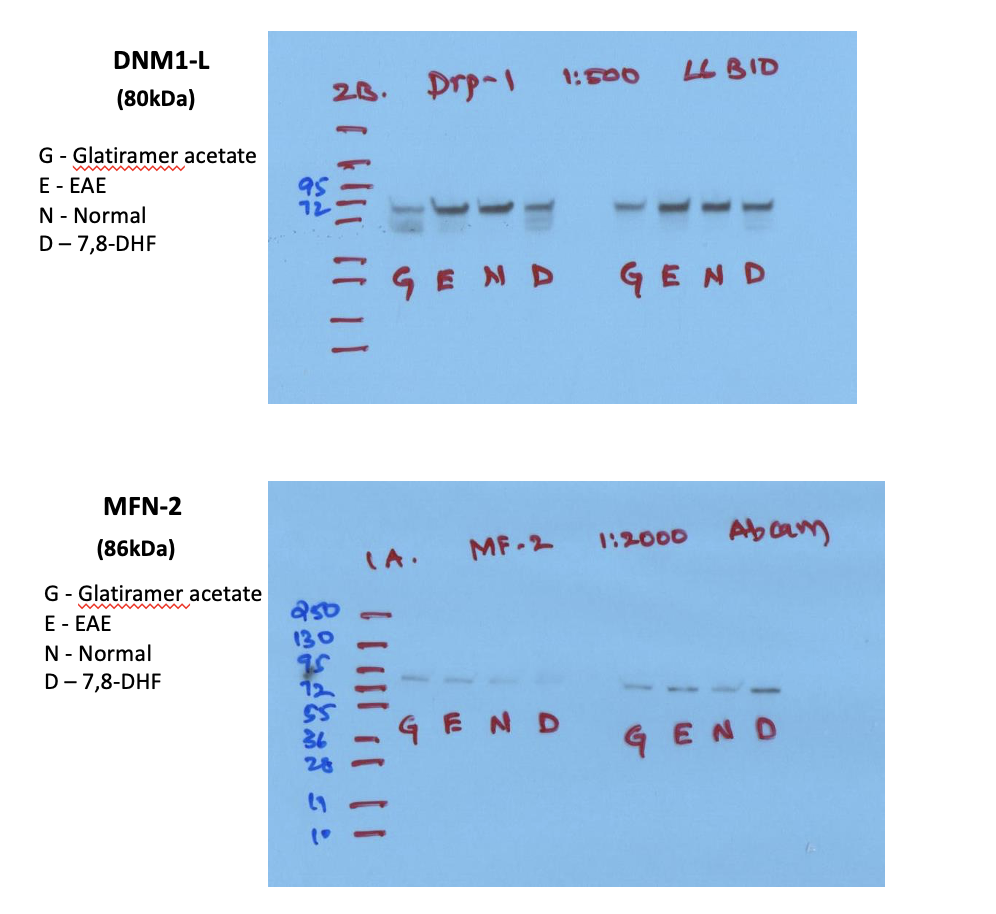

Supplement: Supplementary file 1 — Supplementary Information. [file 41598_2023_29852_MOESM1_ESM.docx]
